# Preoperative Embolization Facilitates Segmental Resection of Pulmonary Sequestration in an Infant

**DOI:** 10.1055/s-0040-1721043

**Published:** 2021-01-27

**Authors:** Dilan Prasad, Christopher Pennell, Lindsay Grier Arthur, Rajeev Prasad

**Affiliations:** 1 Department of Pediatric General, Thoracic, and Minimally Invasive Surgery, Saint Christopher's Hospital for Children, Philadelphia, Pennsylvania, United States

**Keywords:** pulmonary sequestration, transcatheter arterial embolization, congenital lung malformation

## Abstract

The most common congenital lung malformations are congenital pulmonary airway malformations and pulmonary sequestrations. Many surgeons advocate resection to prevent complications of infection, malignancy, and pneumothorax. The standard of care is lobectomy, but segmentectomy and embolization alone have been reported. These methods avoid the complications of lobectomy but are not widely practiced due to concerns about incomplete resection or involution of the lesion. We present a novel approach to the treatment of a pulmonary sequestration in a 7-month-old male using preoperative embolization followed by a sublobar pulmonary resection. The embolization clearly demarcated the affected lung intraoperatively, thereby facilitating complete removal of the lesion with a segmental lung resection rather than complete lobectomy.

## Introduction

Congenital lung malformations (CLMs) comprise an array of lesions, the most common of which are congenital pulmonary airway malformations (CPAM) and pulmonary sequestrations (PS). Overall, their incidence has been increasing with published rates ranging from one in 2,000 to one in 11,000 births. 1 2 The reason for this increase is not clear but is believed to be due in part to increased neonatal screening ultrasonography. PSs account for up to one-fourth of all lesions. 3 The pathognomonic feature of sequestrations that distinguishes them from the more common CPAM is a systemic arterial supply, usually arising from the aorta. Regardless of the type of CLM, resection is advocated by many to avoid complications of infection, pneumothorax, or malignancy. 4

The standard of care for resecting CLMs is complete lobectomy which ensures the entire lesion is removed. Early resection before symptoms develop is preferred because surgery at that time carries a lower rate of complications. 5 In an effort to avoid the morbidity of lobectomy, authors have attempted segmental pulmonary resections 1 and therapeutic embolization to induce necrosis. 6 Widespread use of these less invasive therapies, however, has been limited because of concerns of incomplete resection or involution. 6 7

We present the case of a 7-month-old boy treated with a hybrid approach using preoperative embolization followed by segmentectomy. The embolization clearly demarcated the intralobar sequestration, thereby aiding intraoperative localization and facilitating complete resection of only the affected portion of lung.

## Case Report

A 3-week-old male was referred to pediatric surgery for evaluation of a congenital lung lesion. He was born at 37 weeks via cesarean section for breech presentation with a birthweight of 2.78 kg. After birth, he developed respiratory distress and was admitted to the neonatal intensive care unit where a chest X-ray demonstrated multiple cystic lucencies in the right lung field initially concerning for a CPAM. Prenatal care was limited and it was unknown if the lesion had been previously diagnosed on ultrasound. He required CPAP for respiratory support which was weaned off on day 1 of life. He was discharged home on day 7 of life to follow up with pediatric surgery.

**Fig. 1:**
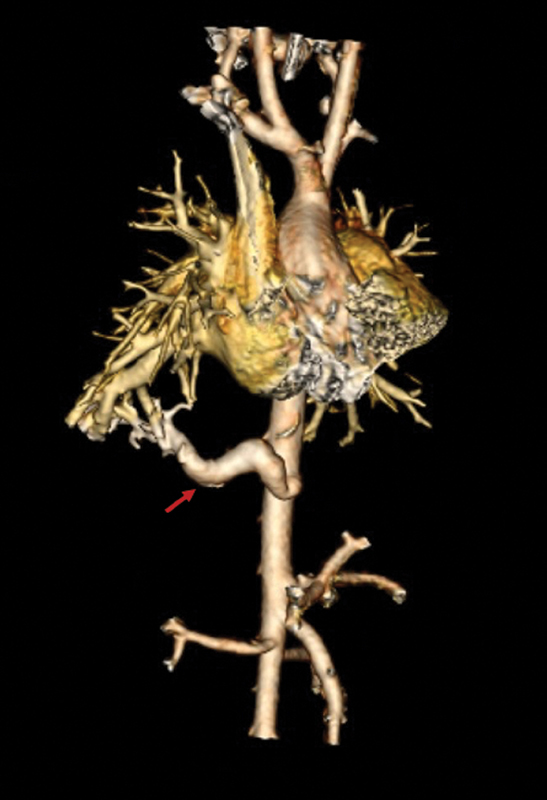
Computed tomography reconstruction demonstrating the vascular anatomy of the pulmonary sequestration. Feeding artery noted by red arrow.

He presented for surgical consultation at 3 weeks of life. Examination revealed a well-appearing 3.75 kg infant, with clear breath sounds bilaterally and no evidence of heart failure. His parents reported no further respiratory symptoms. The computed tomography (CT) angiogram was obtained which revealed a cystic lung lesion in the right lower lobe with a large systemic artery originating from the distal thoracic aorta proximal to the origin of the celiac artery ( Fig. 1 ). These findings confirmed the diagnosis of a PS. The need for surgery to avoid complications related to the sequestration was discussed with the infant's parents. Due to the large size of the systemic feeding vessel, we elected to perform angiographic embolization preoperatively to reduce the risk of intraoperative bleeding. Ideally, we would have performed resection at 3 months of age, but the procedure was delayed until 7 months due to social circumstances beyond our control. During that time, the infant remained asymptomatic.

**Fig. 2:**
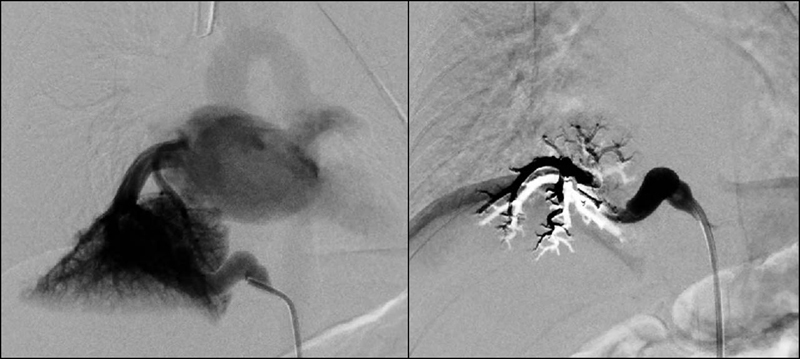
Pre- and postembolization angiogram demonstrating cessation of flow through the pulmonary sequestration.

He was admitted and underwent angiography via right femoral arterial access with interventional radiology. This demonstrated rapid flow through the feeding artery into the sequestration with brisk drainage into the right inferior pulmonary vein. Onyx 18 (ethylene vinyl alcohol, dimethyl sulfoxide) was chosen for embolization because it is a pliable copolymer that would not interfere with the function of a LigaSure device or endothoracic stapler during surgery. We used an occlusion balloon to arrest flow within the feeding vessel so that the brisk arterial flow would not flush the copolymer through the lesion into the pulmonary vein while it hardened. Following embolization, there was no further flow through the sequestration; however, there was some flow noted proximally within the feeding vessel itself ( Fig. 2 ). We were unable to embolize the entire length of the feeding artery because the occlusion balloon could not be placed more proximally in the vessel without risking inadvertent embolization into the aorta. Following this procedure, he was monitored in the PICU overnight before proceeding with surgery the following day.

Thoracoscopy was performed via a standard three-port approach with the infant in the left lateral decubitus position. Upon entry, the large systemic feeding artery itself was not thrombosed. However, the embolized portion of sequestered lung was clearly demarcated and appeared dark purple compared with the surrounding, well-perfused pink lung parenchyma. The inferior pulmonary ligament was mobilized allowing complete visualization of the embolized tissue within the right lower lobe. We had initially planned to perform lobectomy, but after this mobilization, it was clear that the lesion was peripheral and could be fully excised with a nonanatomical segmentectomy. The feeding vessel was mobilized circumferentially and divided using a LigaSure device. The lung parenchyma was similarly divided taking care to include a margin of well-perfused lung around the sequestration. A 16 Fr chest tube was placed and the lung re-expanded with no air leak noted. He was extubated and transferred to the surgical ward without complication. His chest tube was removed on postoperative day 1, and he was discharged on postoperative day 2.

The infant was seen in follow-up 2 weeks after surgery. His parents reported that his activity level was back to normal and denied pain or respiratory symptoms. Final pathology of the specimen confirmed an intralobar PS.

## Discussion

Lobectomy is currently the gold standard for the treatment of CLMs. Increasingly, this is performed thoracoscopically, with excellent results reported in infants. 8 Some authors have advocated observation of these lesions with resection only performed for children that develop pneumonia or other symptoms. A recent meta-analysis compared these approaches for 168 children, 70 of whom had early surgery and 98 of whom were observed and had delayed surgery only after symptoms developed. Of the 98 patients treated expectantly, 64.3% developed symptoms and underwent surgery. The morbidity of resection in this symptomatic group was 31.8%, compared with 10% in the 70 patients who elected to have early resection. 5 Because of this difference in complications, many surgeons advocate for early lobectomy as the standard of care.

Advocates of the resection of asymptomatic lesions also cite a risk of malignant degeneration of CLMs. There is no high quality data quantifying this risk in part due to a lack of life-long follow-up of children with unresected lesions. In addition, many lesions are resected, so it is unknown whether they would ultimately progress to malignancy. Proponents of resection for the prevention of malignancy cite an association of pleuropulmonary blastoma (PPB) with cystic lung lesions. One series of 129 CLMs found that PPB occurred in 2% of lesions previously diagnosed as CPAMs. 9 Conversely, 66% of all PPBs occur within cystic lung lesions. 10

In an effort to reduce the morbidity of lobectomy, authors have reported various lung-preserving techniques including segmental resection or embolization alone to induce necrosis and involution. A segmentectomy preserves healthy and functioning lung parenchyma; however, it is difficult to ensure all abnormal lung tissue is removed. Bagrodia et al compared segmentectomy with lobectomy by reporting 45 infants undergoing surgery for CLMs (19 segmentectomy and 26 lobectomy). 1 They found no difference in surgical complications, but a considerably shorter length of stay after segmentectomy (2 vs. 7 days). No patient developed recurrent symptoms or had residual disease on chest X-ray over a median follow-up of 9 months (range: 0–97 months). These successful results, however, are not universal. A recent meta-analysis of 328 cases (268 lobectomy and 60 segmentectomy) reported that 15% of patients treated with segmentectomy had residual lesions, compared with 0% of those undergoing lobectomy. 7 This highlights the difficulty of ensuring complete resection of all abnormal tissue when performing segmentectomy.

Other authors have attempted to use embolization alone to induce necrosis and involution of lung malformations. This method can only be used to treat PSs because it requires their characteristic systemic arterial supply but has the potential to eliminate the need for resection entirely. Cho et al compared 73 children, 42 of whom were managed with embolization alone, and 31 with surgical resection. 6 Of the children treated with embolization, only three had complete regression, leaving 39 with residual lesions. In addition, four children (9.5%) had complications related to the embolization including two that developed postprocedural sepsis, one who developed a renal abscess secondary to a thrombus, and another with distal embolization of a coil into the iliac artery. Due to the high rate of incomplete regression and procedural complications, the authors concluded that thoracoscopic resection is preferred over embolization alone for the treatment of PSs

The novel method we present here is a hybrid approach that addresses the challenge of incomplete resection that has hindered widespread adoption of lung-preserving surgery for CLMs. We had initially performed the embolization to reduce the risk of intraoperative bleeding from the feeding artery given its large caliber. Due to the technical challenges encountered during the embolization, however, the feeding artery remained patent. The realization that preoperative embolization might be a solution to the problem of incomplete resection arose from this apparent failure, by having the unanticipated consequence of clearly demarcating the lesion from the surrounding normal lung tissue. We therefore do not advocate embolization for the purpose of bleeding control, rather for its potential to facilitate more limited pulmonary resection in infants with PSs.

This approach has limitations that should be addressed. Because two separate procedures are required, the child is subjected to two general anesthetics, the potential for complications from both procedures and additional radiation resulting from fluoroscopy during embolization. There are only a limited number of small studies reporting the rates of complications from embolization of PS in children, with overall complications ranging from 9.5 to 20%. 6 11 12 The rates of distal embolization, concern for which was a primary contributor to our inability to fully embolize the feeding artery, range from 2.4 to 6.3%. 6 11 The combined risks of embolization and sublobar resection will become clearer and potentially decrease with increasing experience with this technique. It is also possible that in the future both procedures could be performed under a single anesthetic with embolization followed immediately by resection. While CT angiography (CTA) is widely practiced for preoperative evaluation of CLMs, it carries a risk of ionizing radiation. 13 Because embolization also requires radiation, magnetic resonance angiography (MRA) could replace CTA to reduce a child's overall radiation exposure if preoperative embolization is planned. MRA, however, often requires anesthesia, so this potential benefit would need to be weighed against the risks of undergoing an additional general anesthetic early in infancy.

As utilizing this technique requires a systemic arterial supply, its application is limited to PSs, which make up only a subset of CLMs. In addition, there is unlikely to be any significant benefit of preoperative embolization of extralobar sequestrations as these lesions are entirely separate from the normal lung and therefore easily identifiable intraoperatively.

Finally, this report is of a single patient with limited follow-up so the long-term risks and benefits of this hybrid approach are unclear. The IDEAL Collaboration has established recommendations for the development and implementation of new surgical innovations. 14 15 Stage 1 (IDEA) includes a first description of a new technique in humans. This can occur after extensive preoperative planning or due to unforeseen intraoperative circumstances as in our case. Following this, new techniques must be modified, refined, and operator learning curves overcome with experience in an increasing number of cases prior to more widespread use (Stage 2a-Development, Stage 2b-Exploration). It is not until this second stage of development and exploration that long-term outcomes can be assessed.

## Conclusion

We have presented a hybrid approach to the treatment of a PS that used preoperative embolization followed by sublobar resection to preserve lung parenchyma while ensuring adequate resection of the lesion. As a first description, this report should be considered primarily one of technical feasibility. We believe the technical success of this hybrid approach is promising and warrants further investigation in a larger cohort of children to better quantify its risks and potential for success.

## Notes

New Insights and the Importance for the Pediatric Surgeon We present a novel approach to treating pulmonary sequestrations in which a more limited, segmental resection is made feasible by preoperative embolization.
